# Integrating Metabolomics and Machine Learning for Advanced Chemical Detection

**DOI:** 10.3390/s26103001

**Published:** 2026-05-10

**Authors:** Gianfranco Picone

**Affiliations:** Department of Agricultural and Food Sciences (DISTAL), University of Bologna, Piazza Goidanich 60, Cesena FC, 47521 Bologna, Italy; gianfranco.picone@unibo.it; Tel.: +39-0547-338106

**Keywords:** metabolomics, machine learning, chemometrics, chemical detection, pattern recognition, data-driven analysis

## Abstract

Metabolomics has emerged as a powerful analytical approach for comprehensive chemical profiling in complex biological and environmental systems. The increasing volume, dimensionality, and complexity of metabolomics data have driven the adoption of machine learning (ML) techniques to enhance chemical detection, classification, and interpretation. This narrative review critically discusses the integration of metabolomics and machine learning for advanced chemical detection, with particular emphasis on analytical workflows, data preprocessing strategies, supervised and unsupervised learning models, and validation approaches. In this context, advanced chemical detection refers to the data-driven identification, classification, and quantification of chemical signatures in complex matrices with improved sensitivity, selectivity, robustness, and interpretability. Current applications across food science, environmental monitoring, clinical diagnostics, and exposomics are discussed, along with key challenges related to data quality, interpretability, and reproducibility. Finally, future perspectives on explainable AI, multimodal data integration, and standardized pipelines are highlighted.

## 1. Introduction

Metabolomics aims to characterize the complete set of small molecules present in a biological, food, or environmental system, providing a direct snapshot of chemical composition and biochemical activity [1,2,3]. Advances in analytical technologies such as nuclear magnetic resonance (NMR) spectroscopy, gas chromatography–mass spectrometry (GC–MS), liquid chromatography–mass spectrometry (LC–MS), and ion mobility (IM)–based platforms have enabled high-throughput and high-resolution metabolite detection [4,5,6,7]. However, these techniques generate large, highly multivariate datasets that challenge conventional univariate and rule-based analytical approaches. In this review, the term “advanced chemical detection” refers to the ability to identify, classify, and quantify chemical compounds or chemical signatures in complex matrices with enhanced sensitivity, selectivity, robustness, and predictive capability. This concept extends beyond conventional single-analyte detection and includes multivariate, data-driven approaches capable of capturing subtle and complex chemical patterns. In this framework, metabolomics provides comprehensive chemical profiling, whereas machine learning (MI) enables the extraction of predictive and interpretable information from high-dimensional datasets. Their integration therefore supports improved chemical detection in biological, environmental, food, and sensor-based systems.

ML has become an essential component of modern metabolomics, offering powerful tools for pattern recognition, classification, regression, and feature selection in high-dimensional chemical data [7,8,9]. Unlike traditional chemometric methods, ML algorithms can model complex, nonlinear relationships and exploit subtle multivariate signatures associated with specific chemical states or conditions. When appropriately integrated, metabolomics and machine learning enable more sensitive and robust chemical detection, facilitate biomarker discovery, and improve predictive performance across a wide range of applications [10]. Several recent reviews have examined the use of ML in metabolomics and sensing technologies. For instance, it has been reviewed in relation to metabolomics-based disease modeling and classification [11,12,13], while other reviews have focused on ML-assisted biosensors for Alzheimer’s disease [14], ML-assisted sensing techniques for monitoring COVID-19 [15], food-safety biosensing [16], and broader biosensor applications [17]. These studies demonstrate the increasing importance of ML for signal processing, classification, biomarker detection, and decision support. However, most previous reviews have focused either on metabolomics data analysis or on biosensor technologies as separate domains. In contrast, the present review provides an integrated perspective on metabolomics and machine learning for advanced chemical detection, with particular attention to analytical workflows, validation challenges, sensor-based translation, and real-world applicability.

**Figure 1:**
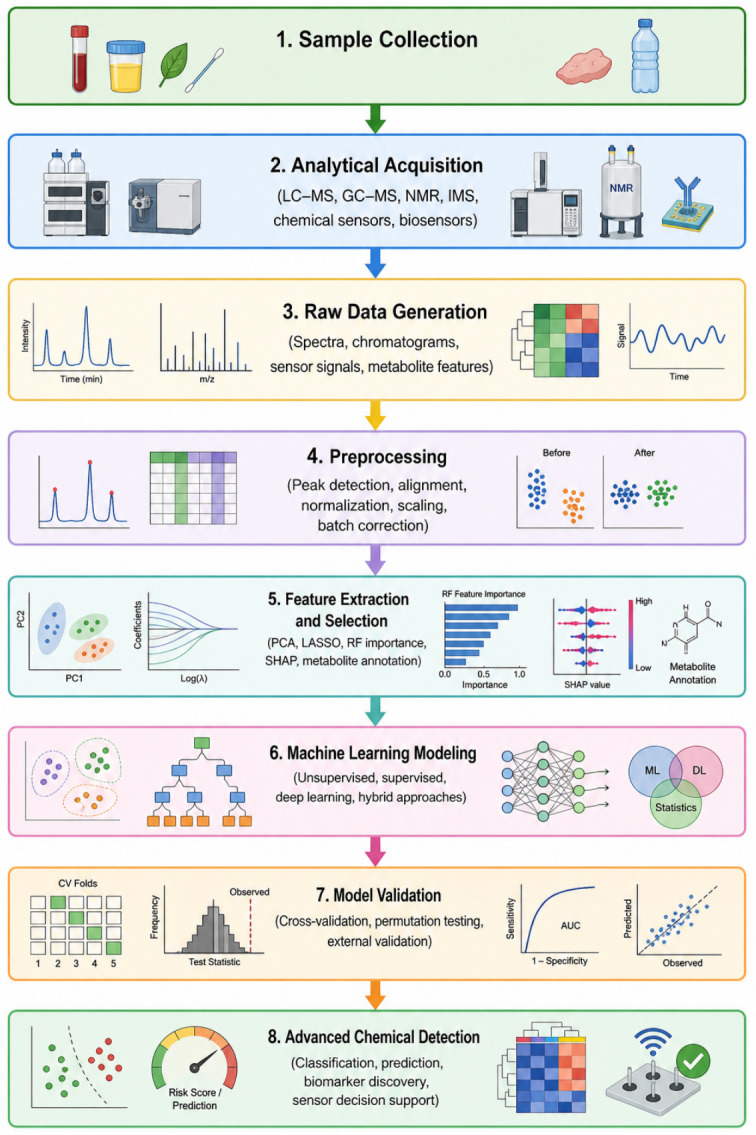
Integrated metabolomics-machine learning workflow for advanced chemical detection. The workflow includes sample collection, analytical acquisition by LC-MS, GC-MS, NMR, ion mobility, or sensor-based platforms, preprocessing, feature extraction and selection, model development, validation, and chemical interpretation. The reliability of the final output depends on data quality, appropriate preprocessing, robust validation, and interpretability of the selected machine learning model.

This review focuses on the integration of metabolomics and ML for advanced chemical detection. First, the main metabolomics platforms and data characteristics relevant to ML-based analysis are summarized. Then, a discussion on common ML strategies, including unsupervised, supervised, and deep learning approaches, is discussed, along with their roles within metabolomics workflows. Applications in food authentication and safety, environmental and chemical exposure monitoring, and clinical diagnostics are also reviewed. Finally, current limitations and outline future directions toward interpretable, reproducible, and application-ready ML-driven metabolomics are outlined (Figure 1).

## 2. Metabolomics Data Characteristics and Analytical Platforms

Metabolomics refers to the systematic identification and quantification of small molecules (<1500 Da) in biological samples such as plasma, urine, tissues, and cell extracts. As metabolites represent the final products of cellular regulatory processes, metabolomics provides a direct snapshot of physiological and pathological states [18]. Unlike genomics and proteomics, which measure potential biological function, metabolomics reflects real-time biochemical activity. The field has grown rapidly due to advances in analytical chemistry, instrumentation sensitivity, computational tools, and bioinformatics infrastructure.

Applications span biomarker discovery, systems biology, toxicology, nutrition, microbiome research, agriculture, and precision medicine [19,20]. Despite this progress, comprehensive metabolome coverage remains challenging due to chemical heterogeneity, concentration variability, and incomplete metabolite annotation.

### 2.1. Chemical Diversity and Structural Complexity

One of the defining features of metabolomics data is the extensive chemical diversity of metabolites. Small molecules vary widely in polarity, molecular weight, solubility, volatility, and functional groups, encompassing classes such as amino acids, lipids, carbohydrates, nucleotides, and secondary metabolites. This structural heterogeneity poses a major analytical challenge, as no single platform can comprehensively detect all metabolite classes [1,19,21,22]. For instance, polar metabolites are more effectively analyzed using LC–MS or capillary electrophoresis–MS (CE–MS) [23], whereas volatile and thermally stable compounds are typically profiled by GC–MS following derivatization [24,25]. Lipidomics, a subfield of metabolomics, often requires specialized LC–MS workflows optimized for hydrophobic compounds [26]. This diversity necessitates the use of complementary analytical techniques and complicates downstream data integration, particularly in ML workflows where feature comparability is essential.

### 2.2. Dynamic Range and Quantitative Variability

Metabolomics datasets are characterized by a wide dynamic range, with metabolite concentrations spanning several orders of magnitude within a single biological sample. Highly abundant compounds, such as sugars and amino acids, coexist with low-abundance metabolites involved in signaling and regulatory pathways, posing significant challenges for accurate detection and quantification. Analytical platforms must therefore combine high sensitivity with broad dynamic range to ensure reliable coverage of both major and trace-level metabolites [27].

In addition to concentration disparities, metabolomics data exhibit considerable quantitative variability arising from both biological and technical sources. Biological variability reflects intrinsic differences among samples, including genetic background, environmental exposure, and physiological conditions. Technical variability is introduced during sample preparation, extraction efficiency, chromatographic separation, and instrumental analysis, as well as through batch effects and signal drift. These sources of variation can lead to systematic and random fluctuations in metabolite intensities, potentially obscuring true biological differences if not properly controlled. The combined effects of dynamic range and quantitative variability have important implications for downstream statistical analysis and ML applications. Without appropriate preprocessing, highly abundant metabolites may dominate the data structure, while low-intensity but biologically relevant features may be underrepresented. To address these issues, normalization strategies, scaling approaches (e.g., autoscaling), and data transformations such as logarithmic conversion are commonly applied to stabilize variance and improve comparability across samples [28]. These preprocessing steps are essential to enhance model robustness, reduce bias, and ensure the reliable extraction of meaningful biochemical information.

### 2.3. Missing Data and Sparsity

Missing data and sparsity are inherent characteristics of metabolomics datasets and represent significant challenges for data analysis and ML applications. Missing data refer to the absence of measured values for specific metabolites across samples, which can arise from various sources, including limits of detection, ion suppression, peak misalignment, and inconsistencies in signal acquisition [29]. Missingness may be classified as missing completely at random (MCAR), missing at random (MAR), or missing not at random (MNAR), with the latter often associated with metabolites present at concentrations below the detection threshold [30]. Sparsity, on the other hand, describes the presence of a high proportion of zero or near-zero values within the data matrix, resulting in incomplete metabolite profiles across samples. This phenomenon is particularly common in untargeted metabolomics, where not all metabolites are consistently detected in every sample due to biological variability and analytical limitations [31].

Both missing data and sparsity can adversely affect statistical inference and ML model performance by reducing data completeness, introducing bias, and distorting variance structure. To address these issues, appropriate data preprocessing strategies are required, including imputation methods such as k-nearest neighbors, multiple imputation, and machine learning-based approaches (e.g., random forest imputation), as well as feature filtering and transformation techniques [29,32]. Careful handling of missingness is essential to preserve biological relevance, improve model robustness, and ensure reliable interpretation of metabolomics data.

### 2.4. Technical Variability and Batch Effects

Technical variability and batch effects represent major sources of non-biological variation in metabolomics datasets and can significantly compromise data quality and interpretability. Technical variability refers to fluctuations in measured metabolite intensities that arise from experimental and analytical procedures rather than true biological differences. These variations may originate from multiple stages of the workflow, including sample collection, storage conditions, extraction protocols, chromatographic separation, ionization efficiency, and instrument performance [5,33,34,35]. Such variability may arise from differences in extraction efficiency, chromatographic performance, ionization conditions, and instrument drift over time. Batch effects constitute a specific form of technical variability and occur when samples are processed or analyzed in different groups (batches) under slightly varying conditions. Even when protocols are standardized, subtle differences in instrument calibration, reagent lots, environmental conditions, or acquisition time can introduce systematic shifts between batches. As a result, samples analyzed within the same batch tend to be more similar to each other than to samples from different batches, regardless of their biological origin. This can lead to artificial clustering patterns and confounding effects that obscure true biological signals.

In metabolomics studies, batch effects are particularly problematic due to the high sensitivity of analytical platforms such as LC–MS and GC–MS [36,37,38]. Instrumental drift over time, changes in detector response, and variations in ionization efficiency can progressively alter signal intensities, leading to time-dependent biases. Without proper correction, these artifacts may dominate the data structure and significantly affect downstream statistical analysis and machine learning performance. To address technical variability and batch effects, rigorous quality control (QC) strategies are essential. The use of pooled QC samples, injected periodically throughout the analytical run, allows monitoring of instrument stability and signal drift. Internal standards are also employed to correct for variability in extraction and ionization efficiency. In addition, data-driven correction methods, such as empirical Bayes approaches (e.g., ComBat), LOESS-based signal correction, and other normalization techniques, are widely used to remove systematic batch-related variation [39].

From a machine learning perspective, the presence of uncorrected batch effects can lead to biased models that learn technical artifacts instead of biologically meaningful patterns. This reduces model robustness, limits external validation, and compromises the reproducibility of results. Therefore, effective batch correction and data harmonization are critical prerequisites for reliable ML-based metabolomics analysis, ensuring that predictive models capture true biochemical variation rather than experimental noise.

### 2.5. Noise, Signal Overlap, and Data Preprocessing

Metabolomics data generated by analytical platforms such as MS and NMR are inherently complex and often affected by noise and signal overlap, which can compromise data quality and downstream analysis. Noise refers to unwanted random or systematic signals that do not originate from true metabolites but arise from instrumental fluctuations, electronic background, chemical contaminants, or environmental interference [40]. This noise can obscure low-intensity metabolite signals and reduce the sensitivity and reliability of detection.

Signal overlap, also known as peak overlap or spectral convolution, occurs when signals from different metabolites partially or fully coincide within the same spectral or chromatographic region. This is particularly common in complex biological samples, where thousands of compounds may co-elute or produce similar mass-to-charge (m/z) ratios or resonance frequencies [41]. In MS-based metabolomics, co-eluting compounds and isobaric species can generate overlapping peaks, while in NMR spectra, signals from structurally related metabolites may share similar chemical shifts. As a result, distinguishing and accurately quantifying individual metabolites becomes challenging.

To address these issues, comprehensive data preprocessing is required as a critical step in metabolomics workflows. Data preprocessing encompasses a series of computational procedures designed to enhance signal quality, reduce technical variability, and improve comparability across samples [42,43,44]. Key steps include peak detection (identifying true metabolite signals), deconvolution (separating overlapping peaks), retention time alignment (correcting shifts across runs), normalization (adjusting for systematic variation), and scaling (ensuring balanced feature contribution) [45]. The quality of preprocessing directly influences downstream statistical and ML analyses. Inadequate handling of noise and signal overlap can lead to inaccurate feature extraction, biased models, and reduced reproducibility. Conversely, well-optimized preprocessing pipelines improve signal-to-noise ratio, enhance feature consistency, and facilitate more reliable pattern recognition and predictive modeling. Recent developments have increasingly incorporated machine learning and deep learning techniques into preprocessing workflows. These approaches enable automated peak detection, improved deconvolution of overlapping signals, and adaptive noise filtering, thereby reducing operator-dependent variability and enhancing analytical robustness [46]. Consequently, data preprocessing represents a crucial interface between raw analytical output and advanced computational analysis, particularly in ML-driven metabolomics studies.

### 2.6. Analytical Platforms for Metabolomics

The comprehensive characterization of the metabolome relies on advanced analytical platforms capable of detecting and quantifying chemically diverse metabolites across a wide dynamic range. As discussed in the previous sections, the intrinsic complexity of metabolomics data—including chemical heterogeneity, variability, noise, and missing values—places significant demands on analytical technologies. No single platform is sufficient to capture the full spectrum of metabolites present in biological, food, or environmental samples. Consequently, metabolomics studies typically employ complementary techniques to achieve broader coverage and improve analytical reliability.

Among the available platforms, mass spectrometry MS and NMR spectroscopy represent the most widely used and established approaches, each offering distinct advantages and limitations. In addition, emerging technologies such as ion mobility spectrometry and imaging mass spectrometry are expanding analytical capabilities by providing additional dimensions of separation and spatial information. The selection of an appropriate analytical platform depends on several factors, including the physicochemical properties of target metabolites, sensitivity requirements, sample type, and the specific objectives of the study. Importantly, the performance and output of these analytical platforms directly influence downstream data processing and machine learning applications. Therefore, understanding their principles, strengths, and limitations is essential for designing robust metabolomics workflows and ensuring accurate chemical detection.

#### 2.6.1. Mass Spectrometry (MS)-Based Platforms

MS represents the most widely used analytical approach in metabolomics due to its high sensitivity, selectivity, and broad metabolite coverage. LC–MS is extensively applied in untargeted metabolomics, enabling the detection of a wide range of metabolites with varying polarity. GC–MS offers high chromatographic resolution and reproducibility, supported by well-established spectral libraries, although it requires derivatization for non-volatile compounds. CE–MS is particularly suited for the analysis of polar and charged metabolites. Despite these advantages, MS-based techniques are subject to limitations, including ion suppression, matrix effects, and variability in ionization efficiency, which can affect quantitative accuracy and reproducibility.

#### 2.6.2. Nuclear Magnetic Resonance (NMR) Spectroscopy

NMR spectroscopy provides a complementary analytical platform characterized by high reproducibility, minimal sample preparation, and inherent quantitative capability. Although less sensitive than MS-based approaches, NMR offers robust and highly reproducible measurements, making it particularly suitable for longitudinal and clinical studies. Additionally, NMR provides valuable structural information that facilitates metabolite identification.

#### 2.6.3. Ion Mobility Spectroscopy (IMS) and Emerging Technologies

IMS, often coupled with MS, introduces an additional dimension of separation based on molecular shape, size, and charge, improving metabolite resolution and aiding in the discrimination of isomeric compounds. Emerging technologies, including imaging mass spectrometry and ambient ionization techniques, further expand metabolomics capabilities by enabling spatially resolved and in situ analyses.

The integration of these advanced platforms enhances metabolome coverage but also increases data complexity and heterogeneity, reinforcing the need for robust computational and machine learning approaches for data integration and interpretation.

## 3. Machine Learning Strategies in Metabolomics

The intrinsic complexity and high dimensionality of metabolomics data necessitate the use of advanced computational approaches for effective interpretation. As discussed in previous sections, metabolomics datasets are characterized by chemical diversity, wide dynamic range, missing values, technical variability, and data heterogeneity. These factors limit the applicability of traditional univariate and linear statistical methods, particularly when addressing multivariate biochemical systems. In this context, ML has emerged as a powerful framework for extracting meaningful patterns, reducing dimensionality, and enabling predictive modeling in metabolomics datasets [12,47,48,49,50].

ML approaches are particularly well-suited to capture nonlinear relationships and complex multivariate interactions inherent to metabolomics data. By integrating information derived from diverse analytical platforms, ML facilitates improved feature selection, classification, and chemical detection. Moreover, ML methods support both hypothesis-driven and data-driven analyses, enabling the identification of hidden structures and predictive biomarkers. Depending on the availability of labeled data and the analytical objectives, ML methods can be broadly categorized into unsupervised, supervised, and deep learning approaches, each playing a distinct role within metabolomics workflows [51,52].

### 3.1. Unsupervised Learning

Unsupervised machine learning methods (UMLM) are widely used for exploratory data analysis, dimensionality reduction, and pattern discovery in metabolomics datasets [53,54]. These approaches operate without predefined class labels and aim to uncover intrinsic structures within the data. Principal component analysis (PCA) is one of the most commonly applied techniques in metabolomics [55,56]. PCA reduces data dimensionality by transforming original variables into a smaller set of orthogonal components that capture the maximum variance [57]. This enables visualization of sample distributions, identification of trends, and detection of outliers. PCA is frequently used as a first step in metabolomics workflows to assess data quality and identify potential confounding factors such as batch effects [58]. Clustering techniques, including hierarchical clustering and k-means, are also widely employed to identify natural groupings of samples or metabolites based on similarity measures [59]. These methods are useful for detecting patterns associated with biological conditions, environmental exposure, or sample classes [60]. However, clustering results can be sensitive to distance metrics and scaling methods, requiring careful preprocessing. While unsupervised methods provide valuable insights into data structure and variability, they do not directly support predictive modeling. Nevertheless, they play a critical role in feature exploration and hypothesis generation, forming the foundation for subsequent supervised analyses.

### 3.2. Supervised Learning

Supervised machine learning methods (SMLM) are central to classification and regression tasks in metabolomics, where models are trained using labeled datasets to predict outcomes or assign samples to predefined classes [12]. These approaches are extensively applied in biomarker discovery, disease classification, food authentication, and chemical detection. Random forest (RF) is a widely used ensemble learning method that constructs multiple decision trees and aggregates their predictions [61]. RF is particularly robust to noise, capable of handling high-dimensional data, and provides measures of variable importance, making it well-suited for metabolomics applications. Support vector machines (SVMs) are effective in modeling complex nonlinear relationships through kernel functions. SVMs are particularly advantageous in high-dimensional spaces and are commonly used for classification tasks in metabolomics studies [62,63,64]. Partial least squares discriminant analysis (PLS-DA), a supervised extension of PLS regression, is widely applied in metabolomics for classification and feature selection [65,66]. Although PLS-DA is popular due to its interpretability and ability to handle collinear variables, it is prone to overfitting and requires rigorous validation strategies such as cross-validation and permutation testing [67].

Overall, supervised learning methods enable the identification of metabolite signatures associated with specific biological or chemical conditions, supporting both predictive modeling and mechanistic interpretation.

### 3.3. Deep Learning Approaches

Deep learning (DL) techniques have recently gained increasing attention in metabolomics due to their ability to learn hierarchical feature representations directly from raw or minimally processed data [68]. Artificial neural networks (ANNs), convolutional neural networks (CNNs), and autoencoders are among the most commonly applied DL architectures [69]. CNNs are particularly effective for analyzing spectral data, as they can capture local patterns and spatial relationships within mass spectra or NMR signals [70]. Autoencoders are used for dimensionality reduction and feature extraction, enabling the identification of latent representations that capture complex data structures.

DL approaches offer several advantages, including reduced reliance on manual feature engineering and improved performance in large-scale datasets. However, their application in metabolomics remains limited by relatively small sample sizes, high computational requirements, and challenges related to interpretability. The “black-box” nature of DL models can hinder biological interpretation and limit their acceptance in clinical and regulatory settings.

### 3.4. Feature Selection and Model Interpretation

Feature selection is a critical component of metabolomics-based machine learning, aimed at identifying the most informative variables while reducing dimensionality and improving model performance. Given the “large p, small n” nature of metabolomics datasets, effective feature selection is essential to avoid overfitting and enhance model generalizability. Common feature selection techniques include recursive feature elimination, LASSO (least absolute shrinkage and selection operator) regression, and tree-based importance measures derived from models such as random forest [71]. These methods help prioritize metabolites that contribute most significantly to classification or prediction tasks. In parallel, model interpretability has become an increasingly important aspect of ML applications in metabolomics. Techniques such as SHAP (Shapley Additive Explanations) and LIME (Local Interpretable Model-Agnostic Explanations) are used to quantify the contribution of individual features to model predictions [72]. These approaches enhance transparency, facilitate biological interpretation, and support the identification of potential biomarkers. The integration of feature selection and interpretability methods is essential for translating computational results into meaningful biological insights and for improving the reliability and reproducibility of ML-driven metabolomics studies.

### 3.5. Critical Comparison of Machine Learning Approaches in Metabolomics

Although a wide range of machine learning approaches have been applied in Metabolomics, their suitability depends strongly on dataset size, dimensionality, analytical platform, preprocessing strategy, and the intended application. In metabolomics, the common “large p, small n” structure, where thousands of variables are measured in relatively few samples, makes model selection and validation particularly important. Therefore, the best-performing model is not necessarily the most complex one, but rather the model that provides the best balance between predictive performance, robustness, interpretability, and external validity. PCA remains essential for exploratory analysis, outlier detection, visualization, and identification of technical variation such as batch effects. However, PCA is not a predictive classifier and should not be interpreted as evidence of diagnostic or chemical-detection performance. Clustering methods can reveal natural groupings among samples or metabolites, but their results depend strongly on scaling procedures, distance metrics, and the selected number of clusters.

Among supervised methods, RF is widely used because of its robustness to noise, ability to model nonlinear relationships, and capacity to provide variable-importance measures. Nevertheless, RF models may still overfit when the number of samples is small, particularly if feature selection is performed before cross-validation. SVMs are effective in high-dimensional data and can handle nonlinear decision boundaries through kernel functions, but they require careful tuning and are less transparent than linear models. PLS-DA is popular in metabolomics because it is interpretable and handles collinear variables, but it is particularly sensitive to overfitting and requires rigorous cross-validation, permutation testing, and preferably external validation.

Deep learning methods, including artificial neural networks, convolutional neural networks, and autoencoders, can capture complex nonlinear patterns and may be particularly useful for spectral or imaging data. However, their application in metabolomics remains constrained by limited sample sizes, high computational demands, and reduced interpretability. Therefore, deep learning should be applied cautiously unless sufficiently large and diverse datasets are available.

Overall, direct comparisons between machine learning methods remain difficult because published studies often differ in sample size, preprocessing, feature filtering, validation design, and reported performance metrics. Consequently, future studies should prioritize standardized benchmarking, external validation, and transparent reporting rather than relying only on high internal classification accuracy. A critical comparison of commonly used methods is summarized in Table 1.

## 4. Applications in Advanced Chemical Detection

The integration of metabolomics and ML has enabled substantial advances in chemical detection across multiple domains by improving sensitivity, specificity, and data interpretability. The capacity of ML algorithms to extract meaningful patterns from complex, high-dimensional metabolomics datasets has transformed the field from descriptive and exploratory analyses toward predictive, diagnostic, and decision-support frameworks. By leveraging multivariate relationships and nonlinear interactions, ML enhances the detection of subtle metabolic perturbations that may not be captured using conventional statistical approaches [68,73]. In particular, ML-driven metabolomics enables the identification of latent biochemical signatures associated with specific physiological states, environmental exposures, or food matrices. These capabilities support high-throughput screening, automated classification, and real-time decision-making. Within the emerging Foodomics framework, the integration of advanced analytical platforms such as NMR with machine learning has been shown to provide powerful tools for modeling food–human interactions, identifying dietary biomarkers, and understanding metabolic responses to food intake [74,75]. For example, studies have demonstrated the application of combined GC–MS and NMR metabolomics, together with multivariate analysis, to identify food intake biomarkers (e.g., dairy-related metabolites such as lactose-derived compounds and microbial metabolites), highlighting the potential of metabolomics–ML approaches for nutritional assessment and personalized diet evaluation [76,77]. Here are some examples of applications in Advanced Chemical Detection.

To improve the readability of this section and to facilitate comparison across application areas, representative uses of ML-driven metabolomics in advanced chemical detection are summarized in Table 2.

### 4.1. Biomedical Diagnostics

In biomedical research, ML-driven metabolomics has been widely applied for the identification of disease-specific metabolic signatures, enabling early diagnosis, disease classification, and prognosis. Metabolic reprogramming is a hallmark of many pathological conditions, including cancer, diabetes, cardiovascular diseases, and neurodegenerative disorders. ML algorithms can detect subtle alterations in metabolic pathways, allowing discrimination between healthy and diseased states with high accuracy [78]. For example, metabolomics combined with ML has been used to identify panels of metabolites associated with early-stage cancers, such as prostate, breast, and colorectal cancer, enabling non-invasive diagnostics based on biofluids such as plasma, urine, or saliva [79]. Similarly, in neurodegenerative diseases such as Alzheimer’s disease, ML models applied to metabolomics data have revealed altered lipid and energy metabolism pathways, supporting early detection and disease stratification [80]. In addition, ML-driven metabolomics has been applied to metabolic disorders such as type 2 diabetes, where predictive models based on metabolite profiles can identify individuals at risk before clinical onset [81]. These approaches contribute to precision diagnostics and support the development of personalized treatment strategies by linking metabolic phenotypes to disease progression and therapeutic response.

### 4.2. Environmental and Toxicological Analysis

Metabolomics combined with ML plays a critical role in environmental monitoring and toxicology by enabling the detection of biochemical responses to chemical exposure. Environmental pollutants, xenobiotics, and toxic compounds induce measurable perturbations in metabolic pathways, which can be captured as “metabolic fingerprints.” ML algorithms facilitate the classification of exposure scenarios and the identification of specific chemical stressors by analyzing complex metabolomic profiles. For instance, metabolomics–ML approaches have been used to assess exposure to heavy metals, pesticides, and air pollutants, revealing characteristic metabolic alterations related to oxidative stress, inflammation, and energy metabolism [82]. In ecotoxicology, ML models applied to metabolomics data have been used to evaluate the impact of contaminants on aquatic organisms, enabling the assessment of ecosystem health and pollutant toxicity [83,84]. Moreover, these approaches have been explored for the detection of exposure to chemical warfare agents and industrial toxins, where rapid and sensitive identification of biochemical changes is essential for risk assessment and response [85]. Overall, ML-enhanced metabolomics provides a powerful framework for linking chemical exposure to biological effects, supporting both environmental surveillance and toxicological evaluation.

### 4.3. Food Authenticity and Safety

In food science, the integration of metabolomics and ML has significantly enhanced the detection of food adulteration, contamination, and mislabeling. Food products exhibit complex chemical compositions influenced by factors such as raw materials, processing methods, geographical origin, and storage conditions. Metabolomic profiling enables the generation of detailed chemical fingerprints that can be used for authentication, quality assessment, and traceability [44,86]. ML algorithms facilitate the classification of food samples and the detection of subtle compositional differences associated with fraud or quality degradation. For example, ML models applied to metabolomics data have been used to distinguish authentic products from adulterated ones in commodities such as olive oil, wine, honey, and dairy products [87,88,89]. In these cases, metabolite patterns serve as reliable indicators of origin and authenticity. In addition, recent studies within the foodomics framework have highlighted the potential of combining metabolomics with multivariate and ML approaches to characterize food composition and identify dietary biomarkers [90,91]. For instance, Trimigno et al. demonstrated the integration of NMR-based metabolomics and chemometric analysis to investigate food–human interactions and identify biomarkers related to dairy product consumption, providing valuable insights into nutritional assessment and food traceability [76,77]. Furthermore, metabolomics–ML approaches have been applied to detect contaminants such as mycotoxins, pesticide residues, and processing-induced compounds, improving food safety monitoring [92]. These techniques are increasingly integrated into quality control systems, regulatory frameworks, and traceability platforms, supporting transparency and consumer protection in the food supply chain. Overall, the combination of metabolomics and ML represents a powerful strategy for comprehensive food characterization, enabling both authenticity verification and the assessment of food–health relationships [93].

### 4.4. Drug Discovery and Precision Medicine

Metabolomics and ML are also transforming drug discovery and precision medicine by enabling the characterization of metabolic responses to therapeutic interventions [94,95]. Drug administration often induces complex metabolic changes that reflect mechanisms of action, efficacy, and toxicity. ML models applied to metabolomics data can identify metabolic biomarkers associated with drug response, enabling the prediction of therapeutic outcomes and adverse effects. For example, pharmacometabolomics studies have demonstrated that baseline metabolic profiles can predict patient response to specific treatments, such as chemotherapy or cardiovascular drugs [96,97]. In drug development, metabolomics–ML approaches are used to evaluate drug safety and toxicity by identifying early metabolic perturbations associated with adverse effects [98]. This supports more efficient screening of candidate compounds and reduces the risk of late-stage failure. In the context of precision medicine, integrating metabolomics data with ML enables patient stratification based on metabolic phenotype, allowing the selection of tailored therapeutic strategies. This approach supports personalized healthcare by linking individual metabolic profiles to optimal treatment pathways. Collectively, these applications demonstrate the potential of ML-driven metabolomics as a powerful tool for sensitive, accurate, and high-throughput chemical detection. By combining advanced analytical platforms with data-driven modeling, this integrated approach enables deeper insights into complex chemical systems and supports innovation across biomedical, environmental, and industrial domains.

### 4.5. Sensor-Based and Portable Chemical Detection

Beyond conventional laboratory-based metabolomics platforms, the integration of metabolomics and ML is increasingly relevant for advanced chemical sensors and biosensors [99,100,101]. Modern sensing systems, including electrochemical sensors, optical biosensors, electronic noses, electronic tongues, wearable sensors, and microfluidic devices, generate multidimensional signals that can benefit from ML-based preprocessing, pattern recognition, and decision support [100,102,103]. In this context, ML does not replace the sensing element but enhances the interpretation of complex signals, especially when chemical responses are weak, overlapping, or affected by matrix effects [104].

Microelectrode and nanoelectrode arrays represent particularly promising platforms for high-sensitivity and spatially resolved chemical detection [105]. These devices can support single-analyte or multiplexed detection and can generate dynamic electrochemical fingerprints. When combined with metabolomics-inspired feature extraction and ML, such platforms may improve selectivity and enable real-time classification of chemical states [106]. For example, PCA can be used to reduce dimensionality and visualize sensor-response patterns, while supervised models such as SVM, RF, or neural networks can classify samples or predict analyte concentrations [8,55,57,107].

Hybrid PCA-ML frameworks are especially useful when sensor data are high-dimensional, but sample numbers are limited [108]. PCA can reduce noise and collinearity before supervised classification, thereby improving model stability and interpretability. However, PCA-based dimensionality reduction should be applied within the validation loop to avoid information leakage. Bayesian inference and Bayesian inversion approaches also offer important advantages for sensing applications because they allow prior chemical knowledge to be incorporated into the model and provide uncertainty estimates for predicted concentrations or classifications [109,110]. This is particularly relevant in noisy environments, low-concentration detection, and portable sensing systems. Despite these advantages, the translation of ML-enhanced sensors into routine chemical detection remains challenging. Sensor drift, matrix effects, calibration transfer, device-to-device variability, and environmental fluctuations can reduce model robustness [100,104]. Therefore, future studies should include external validation, multi-device testing, real-sample analysis, and long-term stability assessment.

### 4.6. Critical Appraisal of Evidence Quality and Translational Readiness

Although many studies report promising performance of ML-driven metabolomics for chemical detection, the quality of evidence varies considerably across application areas. A recurrent limitation is the reliance on internal cross-validation without independent external datasets. While internal validation is useful for model optimization, it does not fully demonstrate generalizability across laboratories, instruments, populations, or sample matrices. This is particularly important in metabolomics, where preprocessing choices, batch effects, and platform-specific variability can strongly influence model performance. Another important issue is the lack of standardized benchmark datasets. Without common datasets and harmonized reporting criteria, it remains difficult to determine whether one algorithm is genuinely superior to another or whether reported differences result from preprocessing, feature selection, or validation strategy. Moreover, accuracy alone is insufficient to evaluate model quality. For real-world chemical detection, sensitivity, specificity, false-positive rate, calibration performance, robustness to drift, interpretability, and transferability are equally important.

From a translational perspective, the most mature applications are those supported by real-sample analysis, external validation, interpretable biomarkers, and reproducible workflows. In contrast, applications based only on small pilot datasets or single-center studies should be considered exploratory. Therefore, future reviews and experimental studies should move beyond descriptive reporting and assess validation rigor, evidence quality, and practical deployment barriers. A critical appraisal across application areas is summarized in Table 3.

## 5. Challenges and Limitations

Despite significant progress, several challenges continue to limit the full potential of integrating metabolomics and machine learning (ML). One of the primary issues is the imbalance between the number of variables (p) and the number of samples (n), commonly referred to as the “large p, small n” problem. In metabolomics, datasets often contain thousands of metabolite features measured across relatively few samples. This high dimensionality increases the risk of overfitting, where ML models capture noise or dataset-specific patterns rather than generalizable biological signals, ultimately reducing predictive performance on independent datasets [49,111]. Robust validation strategies, such as cross-validation and external validation cohorts, are therefore essential to ensure model reliability. Data heterogeneity further complicates analysis. Metabolomics datasets are inherently influenced by differences in analytical platforms (e.g., LC–MS vs. NMR), sample preparation protocols, instrument settings, and experimental conditions. Such variability leads to inconsistencies in feature detection and quantification across studies. In particular, batch effects, defined as systematic differences introduced during data acquisition across different experimental runs or sample groups, can significantly distort the underlying biological signal if not properly corrected [35,39]. This type of technical variability may result in artificial clustering of samples, thereby misleading statistical analysis and ML model training. Another major limitation is incomplete metabolite annotation, which remains a bottleneck in metabolomics. A substantial proportion of detected features in untargeted metabolomics cannot be confidently identified due to limitations in spectral databases and reference standards. As a result, ML models may rely on unidentified features, making biological interpretation difficult and limiting the translation of computational findings into mechanistic insights [22,112]. In addition, the lack of interpretability of many ML models, particularly deep learning approaches, represents a critical challenge. While complex models such as neural networks can achieve high predictive accuracy, their “black-box” nature makes it difficult to understand how input features contribute to predictions. This lack of transparency hinders the identification of biologically meaningful metabolites and reduces confidence in model outputs, especially in clinical, regulatory, and food safety applications where explainability is essential [72,113]. Recent advances in explainable artificial intelligence (XAI), including SHAP and LIME methods, aim to address this limitation by providing insights into feature importance and model decision processes [114,115]. Finally, the lack of standardized workflows and the limited availability of large, well-curated datasets restrict reproducibility and cross-study comparability. Differences in data acquisition, preprocessing, normalization, and statistical analysis pipelines can lead to inconsistent results across studies. Moreover, the absence of harmonized reporting standards and shared databases limits data integration and meta-analysis efforts [73,116].

Addressing these challenges requires coordinated efforts in methodological standardization, data sharing, and the development of interpretable and robust ML models. Such advances will be essential for improving the reliability, reproducibility, and real-world applicability of ML-driven metabolomics. In real-world applications, several additional constraints limit the deployment of ML-driven metabolomics for chemical detection. First, the lack of standardized benchmarking datasets prevents objective comparisons between algorithms and makes it difficult to determine whether reported performance improvements reflect genuine methodological advantages. Second, model transferability across laboratories remains limited because metabolomics datasets are strongly influenced by analytical platforms, sample preparation protocols, preprocessing workflows, and batch effects. As a result, models trained on one dataset may perform poorly when applied to data generated under different experimental conditions.

Validation rigor is another critical issue. Many studies report high classification accuracy using internal cross-validation, but external validation with independent cohorts or independent analytical batches is less frequently performed. This can result in overly optimistic estimates of performance, particularly when feature selection is conducted before data splitting. To reduce this risk, future studies should use nested cross-validation, permutation testing, independent test sets, and transparent reporting of all preprocessing and modeling steps.

Finally, practical implementation requires more than high predictive accuracy. For routine chemical detection, models must be interpretable, computationally efficient, robust to drift and matrix effects, and compatible with laboratory or sensor workflows. Therefore, future progress should focus not only on developing more complex algorithms but also on improving reproducibility, interpretability, uncertainty estimation, and calibration transfer.

## 6. Future Perspectives and Conclusions

The integration of metabolomics and machine learning (ML) is rapidly evolving, with significant potential to transform chemical detection across biomedical, environmental, and food science domains. Future developments are expected to focus on improving data integration, analytical standardization, and model interpretability, addressing many of the current limitations discussed above. One of the most promising directions is the integration of metabolomics with other omics layers, including genomics, transcriptomics, and proteomics, within a multi-omics framework. This approach enables a more comprehensive systems-level understanding of biological processes and enhances the predictive power of ML models by combining complementary sources of information. In parallel, advances in data fusion strategies and multi-view learning algorithms will facilitate the integration of heterogeneous datasets generated from different analytical platforms. Another key area of development is the advancement of XAI. As ML models become increasingly complex, particularly with the adoption of deep learning approaches, the need for transparency and interpretability becomes critical. XAI methods, such as SHAP and LIME, will play an essential role in linking computational predictions to biologically meaningful insights, thereby increasing trust and facilitating adoption in clinical, regulatory, and industrial contexts. Standardization also represents a major priority for the field. The establishment of harmonized protocols for sample preparation, data acquisition, preprocessing, and reporting will improve reproducibility and enable cross-study comparability. In addition, the expansion of curated metabolite databases and spectral libraries will significantly enhance metabolite identification, addressing one of the main bottlenecks in metabolomics. The increasing availability of large-scale datasets, supported by collaborative data-sharing platforms and open science initiatives, will further improve ML model performance and generalizability. Emerging applications, including real-time metabolomics, integration with wearable technologies, and automated decision-support systems, are expected to expand the practical impact of ML-driven metabolomics. From a sensor-oriented perspective, future developments should prioritize the integration of metabolomics-inspired chemical fingerprinting with portable and miniaturized sensing systems. The combination of microelectrode arrays, nanoelectrode arrays, biosensors, and ML-based signal interpretation could enable real-time and in situ chemical detection. However, successful translation will require robust validation under real operating conditions, standardized reporting of sensor performance, and uncertainty-aware models capable of supporting reliable decision-making.

In conclusion, the integration of metabolomics and machine learning represents a powerful and rapidly advancing paradigm for chemical detection. By combining high-resolution analytical technologies with advanced computational modeling, this interdisciplinary approach enables the extraction of meaningful information from complex biochemical systems. Continued progress in methodological development, interpretability, and standardization will be essential to fully realize the potential of ML-driven metabolomics and to facilitate its translation into real-world applications across science, industry, and healthcare.

## Figures and Tables

**Table 1:** Critical comparison of machine learning methods used in metabolomics-based chemical detection.

Method	Learning Type	Main Strengths	Main Limitations	Validation Requirements	Typical Use in Metabolomics
PCA	Unsupervised	Simple, interpretable, useful for visualization and outlier detection	Not predictive; captures variance, not necessarily class relevance	Assessment of score plots, loading plots, and technical confounders	Exploratory analysis, batch-effect inspection, quality control
Hierarchical clustering/k-means	Unsupervised	Identifies natural sample or metabolite groupings	Sensitive to scaling, distance metrics, and cluster-number selection	Stability analysis and biological plausibility assessment	Sample grouping, metabolite-pattern exploration
PLS-DA	Supervised	Interpretable, handles collinearity, widely used in metabolomics	High risk of overfitting; may generate optimistic classification results	Cross-validation, permutation testing, external validation	Classification, biomarker prioritization
Random Forest	Supervised	Robust to noise, captures nonlinear relationships, provides variable importance	Can overfit small datasets; variable importance may be biased	Nested cross-validation and external validation	Classification, feature ranking, biomarker discovery
SVM	Supervised	Effective in high-dimensional data; suitable for nonlinear classification	Requires parameter tuning; limited interpretability	Hyperparameter optimization and independent validation	Classification of complex metabolomics profiles
Artificial neural networks	Deep learning	Captures nonlinear interactions; flexible model structure	Requires larger datasets; black-box behavior	Large training sets, regularization, external validation	Prediction and classification in large datasets
CNNs	Deep learning	Effective for spectral or image-like data; automatic feature extraction	Computationally demanding; limited interpretability	Independent validation and explainability analysis	Spectral analysis, imaging metabolomics
Autoencoders	Deep learning/representation learning	Useful for dimensionality reduction and latent-feature extraction	Latent features may be difficult to interpret biologically	Reconstruction error assessment and downstream validation	Feature extraction, denoising, data compression

**Table 2:** Representative applications of ML-driven metabolomics in advanced chemical detection.

Application Area	Detection Target	Typical Analytical Platform	Common ML Methods	Main Advantages	Main Practical Limitations
Biomedical diagnostics	Disease-associated metabolite signatures	LC-MS, GC-MS, NMR	RF, SVM, PLS-DA, neural networks	Early detection, non-invasive biomarker discovery, patient stratification	Limited external validation, small cohorts, biological heterogeneity
Environmental monitoring	Pollutants, xenobiotics, exposure signatures	LC-MS, GC-MS, NMR, sensor arrays	PCA, RF, SVM, clustering	Detection of exposure-related metabolic perturbations	Matrix effects, environmental variability, lack of standardized datasets
Food authenticity and safety	Adulteration, geographical origin, contaminants, spoilage	NMR, LC-MS, GC-MS, electronic nose/tongue	PLS-DA, SVM, RF, hybrid PCA-ML	Rapid classification, traceability, quality control	Product variability, batch effects, calibration transfer
Drug discovery and precision medicine	Drug-response metabolites, toxicity markers	LC-MS, NMR, multi-omics platforms	RF, SVM, DL, feature-selection models	Mechanistic insight, toxicity prediction, patient stratification	High cost, limited cohort size, regulatory requirements
Sensor-based chemical detection	Single analytes, multiplexed analytes, sensor fingerprints	Biosensors, electrochemical sensors, micro/nanoelectrode arrays	PCA-ML, Bayesian models, SVM, RF, neural networks	Portable and real-time detection, high-throughput analysis	Signal drift, calibration instability, limited real-world validation

**Table 3:** Critical appraisal of evidence quality and translational readiness across application areas.

Application Area	Typical Evidence Strength	Common Validation Approach	Main Risk of Bias	Translational Readiness	Key Requirement for Improvement
Biomedical diagnostics	Moderate but heterogeneous	Internal cross-validation; limited external validation	Small cohorts, clinical heterogeneity, confounding factors	Medium	Larger multicenter cohorts and external validation
Environmental monitoring	Moderate	Laboratory-controlled validation	Matrix variability and limited field validation	Medium	Real-world environmental sampling and standardization
Food authenticity and safety	Moderate to high for selected products	Cross-validation and occasional external test sets	Product variability, geographical bias, batch effects	Medium-high	Interlaboratory validation and calibration transfer
Drug discovery and precision medicine	Exploratory to moderate	Preclinical or cohort-specific validation	Limited sample size and biological complexity	Medium	Integration with clinical endpoints and multi-omics validation
Sensor-based chemical detection	Exploratory to moderate	Laboratory calibration and classification testing	Sensor drift, device variability, overfitting	Low-medium	Long-term stability testing, real-sample validation, multi-device studies

## Data Availability

No new data were created or analyzed in this study. Data sharing is not applicable to this article.

## Abbreviations

The following abbreviations are used in this manuscript:

ANN	Artificial Neural Networks
CE–MS	Capillary Electrophoresis–Mass Spectrometry
CNN	Convolutional Neural Networks
DL	Deep Learning
GC–MS	Gas Chromatography–Mass Spectrometry
IM	Ion Mobility
LASSO	Least Absolute Shrinkage and Selection Operator
LC-MS	Liquid Chromatography–Mass Spectrometry
LIME	Local Interpretable Model-Agnostic Explanations
MAR	Missing at Random
MCAR	Missing Completely at Random
ML	Machine Learning
MNAR	Missing Not at Random
NMR	Nuclear Magnetic Resonance Spectroscopy
PLS-DA	Partial Least Squares Discriminant Analysis
QC	Quality Control
RF	Random Forest
SHAP	Shapley Additive Explanations
SMLM	Supervised Machine Learning Methods
SVM	Support Vector Machines
UMLM	Unsupervised Machine Learning Methods
XAI	Explainable Artificial Intelligence
